# Control of glioma cell death and differentiation by PKM2–Oct4 interaction

**DOI:** 10.1038/cddis.2013.561

**Published:** 2014-01-30

**Authors:** M Morfouace, L Lalier, L Oliver, M Cheray, C Pecqueur, P-F Cartron, F M Vallette

**Affiliations:** 1 UMR 892 INSERM, 6299 CNRS, Equipe Labellisée (Ligue contre le Cancer), Nantes, France; 2 Faculté de Médecine, Université de Nantes, Nantes, France; 3 Institut de Cancérologie de l'Ouest, Nantes-Saint Herblain, Nantes, France; 4 CHU Hotel-Dieu, Nantes, France

**Keywords:** apoptosis, cancer stem cells, differentiation, glioma, Oct4, PKM2

## Abstract

Glioma stem cells are highly resistant to cell death and as such are supposed to contribute to tumor recurrence by eluding anticancer treatments. Here, we show that spheroids that contain rat neural stem cells (NSCs) or rat glioma stem cells (cancer stem cells, CSCs) express isoforms 1 and 2 of pyruvate kinase (PKM1 and PKM2); however, the expression of PKM2 is considerably higher in glioma spheroids. Silencing of PKM2 enhances both apoptosis and differentiation of rat and human glioma spheroids. We establish that PKM2 was implicated in glioma spheroid differentiation through its interaction with Oct4, a major regulator of self-renewal and differentiation in stem cells. The small molecule Dichloroacetate (DCA), a pyruvate dehydrogenase kinase inhibitor, increases the amount of PKM2/Oct4 complexes and thus inhibited Oct4-dependent gene expression. Taken together, our results highlight a new molecular pathway through which PKM2 can manage gliomagenesis via the control of glioma stemness by Oct4.

Despite major improvements in our knowledge of the biology of glioma and on the molecular and genetics events implicated in the gliomagenesis, little therapeutic progresses have been made in the past years and the survival median for glioblastoma multiforme (GBM) patients remains low.^1^ In these tumors, only a small percentage of cells have the potential to recreate the original tumor to its full heterogeneity^2^ and these cells share phenotypic traits with normal stem cells, in particular, the capacity to form primary and secondary neurospheres in serum-free medium.^3, 4^ Quite remarkably, these neurospheres exhibit phenotypic and genotypic traits that closely mirror that of the original tumor.^5^ In direct reference to the latter properties, these cells have been named cancer stem cells (CSCs) and numerous reports support their existence in *GBM,* the main form of brain tumors in the adult.^3, 4^ GBM CSCs are very resistant to chemo- and radiotherapy and as such, are thought to be responsible for recurrence of gliomas.^6, 7, 8^ Many efforts are currently underway to find therapies that specifically target these CSCs. One promising strategy is the induction of CSC differentiation, as it has been associated with a reduction in tumor malignancy in animal models.^9, 10, 11, 12^

An alternative strategy to conventional anticancer therapies has been to target the specific metabolism of cancer cells to eliminate the tumor. Most cancer cells exhibit a special glucose metabolism, the Warburg effect or aerobic glycolysis.^13^ Recently, it has become obvious that metabolic alterations are intrinsically involved in tumor growth beyond the mere ATP production through many different mechanisms that provide an advantage to tumors under fast growing or hypoxic conditions.^14^ Pyruvate kinase isoform 2 (PKM2) is a crucial regulator of embryonic and cancer cell metabolism and tumor growth.^15^ PKM2 is also involved in many nonmetabolic roles^16^ and in various cellular functions (for example, phosphorylation of histone H3,^17^ beta catenin transactivation^18^ or antioxidant defense^19^). Growth factor stimulations significantly increase the dimer/tetramer PKM2 ratio in cancer cells and consequently activate the protein kinase activity of PKM2.^20^ Thus, the balance between metabolic and non metabolic PKM2 functions, monitored by the dimer/tetramer and pyruvate kinase (PK)/protein kinase ratio, appears to be instrumental for tumor growth.

The metabolism of CSCs has not been extensively studied. However, it is likely that CSC could have different metabolic profiles depending of their origins and degree of differentiation. We have recently observed that spheroids enriched in CSC were more glycolytic than neural stem cells (NSCs) in adult rat brain, although they did not present any alterations in mitochondrial oxidative phosphorylation.^21^ Similar results have been obtained by Zhou *et al.*^22^ On the other hand, Vlashi *et al.*^23^ found that CSCs rely mainly on mitochondrial respiration but can alternatively use glycolysis. Despite apparent contradictory conclusions, these works pointed out that CSC can alternatively use aerobic glycolysis or mitochondrial respiration metabolisms depending on *in vitro* or *in vivo* experimental or environmental conditions.

In the present work, we examine the contribution of PKM2 in glioma spheroids. We provide direct evidence for another ‘non metabolic' role of PKM2 during glioma differentiation, which occurs through its interaction with Oct4, a major regulator of cell pluripotency.^24, 25^ We also report that a small molecule, the Dichloroacetate (DCA), which has been found to be active against several tumors^26, 27^ induce differentiation through the modulation of PKM2/Oct4 interaction.

## Results

### PKM2 is overexpressed in glioma spheroids and regulates cell death

**Figure 1:**
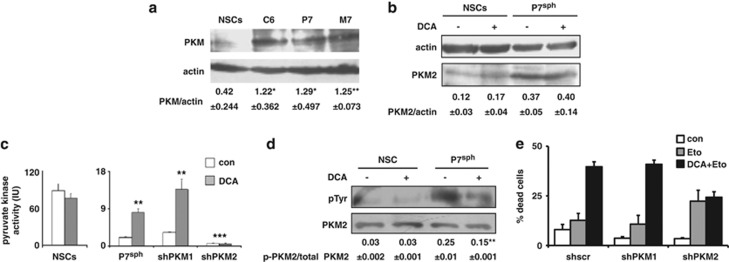
Difference between rat neural and glioma stem cells in the expression of PKM2 and regulation by DCA. (**a**) PKM expression was assessed in rat NSCs or glioma spheroids (sph) by immunoblot using an antibody, which recognizes both M1 and M2 isoforms. The blot shown is representative of three independent experiments. Quantifications are indicated as ratio±S.D. of PKM/actin. *P<0.05; **P<0.01. (**b**) PKM2 expression was assessed in control or DCA-treated (1 mM, 48 h) NSCs or glioma spheroids using a specific anti-PKM2 antibody. The blot shown is representative of three independent experiments. Quantifications are indicated as ratio±S.D. of PKM2/actin. (**c**) The effect of DCA treatment (1 mM, 48 h) on pyruvate kinase activity was measured in NSCs and in glioma spheroids treated with the indicated shRNA designed to target PKM1 or PKM2. **P<0.01; ***P<0.001. (**d**) PKM2 was immunoprecipitated from NSCs or glioma spheroid lysates in control or DCA-treated conditions. The presence of PKM2 and its phosphorylation were determined by anti-phosphotyrosine (pTyr) and anti-PKM2 antibodies, which were detected using western blot. The blot shown is representative of three independent experiments and the values below are the mean±S.D. of the phosphotyrosine intensity determined by densitometry and normalized to total PKM2 intensity (**P<0.01). (**e**) Glioma spheroids treated with sh-scr or shRNA targeting either PKM1 or PKM2 were incubated with etoposide (Eto 50 *μ*g/ml, 12 h) or DCA (1 mM, 48 h)+etoposide. The percentage of dead cells was determined by the Trypan blue dye vital staining

Compared with spheroids that contain rat NSCs, the expression of PKM (analyzed using an antibody that does not discriminate between isoforms 1 and 2) was increased in spheroids that contained CSCs derived from the glioma cell line C6 or from two ethylnitrosourea (ENU)-induced rat gliomas (P7 and M7) obtained as described earlier^21^ (Figure 1a). It should be noted that rat tumors were comparable to high-grade human gliomas.^28^ QPCR analysis of the different PKM isotypes indicated that, compared with NSCs, PKM2 was overexpressed in glioma spheroids, whereas the expression of isoforms 1 pyruvate kinase (PKM1) remained similar (Supplementary Figure S1). This result was confirmed by immunoblots using a homemade antibody (see Materials and Methods) that specifically recognized rat PKM2 (Figure 1b). We have recently shown that DCA induced a metabolic shift in CSCs but not in NSCs.^21^ As shown in Figure 1b, this effect of DCA was not mediated by an increase in the expression of PKM2. We measured the effect of DCA on PK activity in NSCs and glioma spheroids. As illustrated in Figure 1c, DCA did not affect the PK activity in NSCs but significantly increased this activity in glioma spheroids. Note that, despite difference in the expression of the proteins, the basal PK activity was greater in NSCs than in glioma spheroids (Figure 1c). Thus, the larger expression of PKM in glioma spheroids when compared with NSCs may stem from a higher expression of PKM in gliomas in general, not specifically in their CSCs' subpopulation (Supplementary Figure S2). We thus inhibited the expression of PKM1 or PKM2 by RNA interference (Supplementary Figure S3). As shown in Figure 1c, the downregulation of PKM2, but not that of PKM1, abrogated the DCA-induced PK activity in glioma spheroids. On the other hand, it did not affect the PKM activity in NSC (data not shown). We found another difference between cancer and NSCs as PKM2 was phosphorylated in glioma spheroids and this phosphorylation decreased after DCA treatment. Of note, the phosphorylation of PKM2 remained low, almost non-detectable, in NSCs both in the absence and in the presence of DCA (Figure 1d). The latter feature could explain the difference in PK activity between NSCs and glioma spheroids as well as the effect of DCA, as phosphorylated PKM2 has been associated with low active dimers and the absence of phosphorylation with highly active tetramers.^29^

It has been reported that PKM2 was implicated in programmed cell death via the induction of caspases-dependent apoptosis.^30, 31, 32^ As shown in Figure 1e, the knockdown of either PKM1 or PKM2 by shRNAs affected equally but moderately rat glioma spheroids' survival by decreasing spontaneous cell death. However, the silencing of PKM2, but not that of PKM1, induced a nonsignificant increase in Etoposide-induced cell death but completely abolished the DCA-induced potentiation of Etoposide-induced cell death (Figure 1e). Of note, 2-deoxyglucose, a glucose analog and a glycolysis inhibitor, also induced a PKM2-dependent cell death (Supplementary Figure S4).

### DCA-induced glioma spheroids' differentiation *in vitro* and *in vivo*

**Figure 2:**
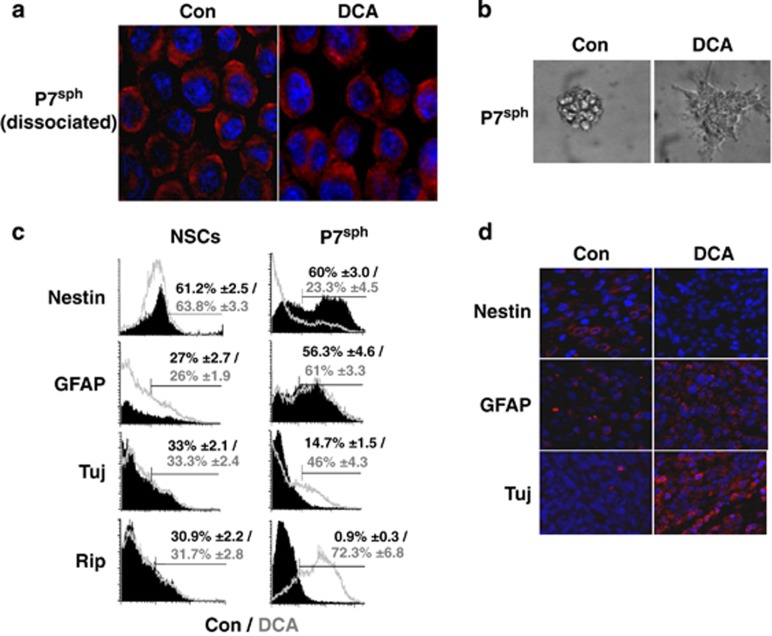
DCA induces glioma spheroids' differentiation *in vitro* and *in vivo*. (**a**) The localization of PKM2 was assessed in dissociated glioma neurospheres by immunostaining and confocal microscopy (blue: DAPI/nucleus; red: PKM2). (**b**) The morphological aspect of glioma neurospheres was shown in control or in DCA-treated conditions (1 mM, 48 h). (**c**) Effect of DCA on neural markers in glioma spheroids and NSCs. The expression of the indicated markers was assessed using flow cytometry. The graphs shown are representative of three independent experiments and the mean±S.D. percentage of positive cells is indicated. (**d**) A total of 5 × 10^4^ glioma spheroids were injected into 22 nude mice, of which 11 were fed with DCA in drinking water as described in Morfouace *et al.*^21^ Tumors were fixed and stained by the indicated antibody (red). Nuclei were stained with DAPI (blue)

Setàk *et al.*^30^ have suggested that the nuclear translocation of PKM2 was important for its pro-apoptotic activity. Under our conditions, PKM2 remained predominantly in the cytoplasm in control cells and no major nuclear translocation was observed upon addition of DCA (Figure 2a). However, the morphological aspect of DCA-treated neurospheres formed from glioma spheroids was drastically different from that of untreated spheroids as most of the cells became adherent, presented an expansive shape and started to form processes after 24 h of treatment (Figure 2b). This morphological modification, which is typical of CSC differentiation,^33^ was not observed in DCA-treated NSC cultures (data not shown).

To evaluate whether DCA has an effect on differentiation, the expression of the stem cell marker nestin and the lineage-specific differentiation markers GFAP (astrocytes), Tuj (neurons) and Rip (oligodendrocyte) were analyzed in control and DCA-treated glioma spheroids and NSCs. As shown in Figure 2c, nestin expression was drastically reduced in DCA-treated glioma spheroids, whereas that of Tuj and Rip was markedly increased upon this treatment. In contrast, the expression of these markers was not altered in NSCs (Figure 2c). Immunocytochemical analyses of the neurospheres confirmed these results (Supplementary Figure S5), suggesting that DCA induced cell differentiation of CSCs contained in glioma spheroids into neural progenitor phenotypes.

We investigated the influence of DCA on the *in vivo* tumor growth and differentiation. Glioma spheroids were injected subcutaneously into nude mice, half of which were fed with DCA *per os* upon injection. As previously described, DCA significantly reduced the growth of rat glioma tumors^21^ (Supplementary Figure S6). At the end of the treatment, tumors were resected, fixed and the expression levels of nestin, GFAP and Tuj were assessed using immunocytochemistry. Consistent with our *in vitro* observation, Nestin expression was undetectable in xenografted tumors after the DCA treatment, whereas those of GFAP and Tuj were increased (Figure 2d). From these results, we conclude that DCA was capable of inducing glioma CSC/glioma spheroid differentiation, both *in vitro* and *in vivo*.

### DCA induces a PKM2–Oct4 interaction and represses Oct4 transcriptional activity that modulates apoptosis

**Figure 3:**
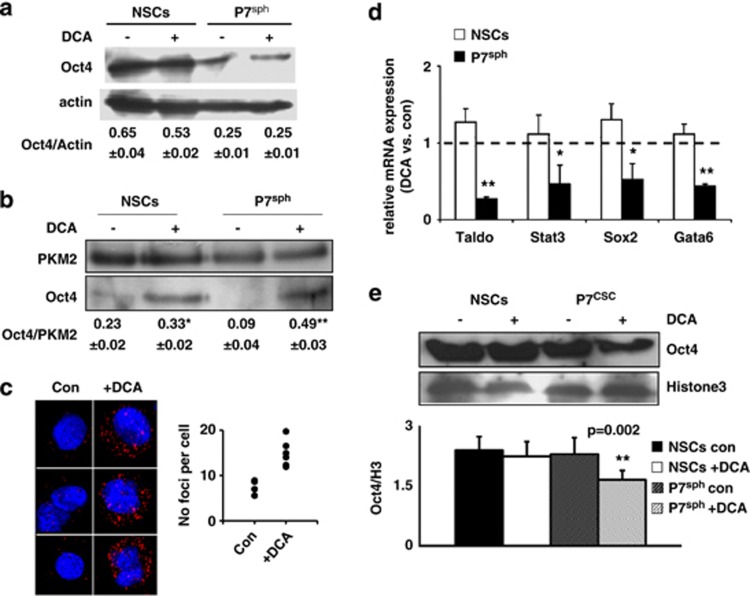
DCA induces PKM2–Oct4 interaction and represses Oct4 transcriptional activity. (**a**) Expression of Oct4 protein in NSCs and glioma spheroids grown as spheroids treated or not with DCA. The protein expression normalized to the expression of actin as indicated in the image as the mean±S.D. from three independent experiments. (**b**) PKM2 was immunoprecipitated from NSCs or glioma spheroids (P7^sph^) as described in experimental procedures. The amount of co-immunoprecipitated Oct4 was revealed by immunoblot, quantified and normalized by PKM2 expression (**P<0.01). (**c**) PKM2–Oct4 complexes (red dots) were visualized by proximity-ligation assay in control or DCA-treated glioma spheroids. Nuclei were stained in blue by DAPI. (**d**) The mRNA expression of four target genes of Oct4 was measured in DCA-treated NSCs or glioma spheroids (P7^sph^) and normalized by their expression in the corresponding control cells (*P<0.05; **P<0.01). (**e**) Chromatin was extracted from NSCs or glioma spheroids. The amount of Oct4 present on the chromatin was revealed by immunoblot, quantified and normalized by the ratio Oct4/H3 calculated (**P<0.01)

Lee *et al.*^34^ have shown that Oct4 could directly interact with PKM2 in embryonic carcinoma cells. We found that Oct4 was overexpressed in NSCs as compared with glioma spheroids and that DCA had no effect on its expression in both cell types (Figure 3a). As DCA promoted CSC differentiation and increased PKM2 activity, we analyzed the role of Oct4–PKM2 interaction upon treatment with DCA. We found that in untreated cells, Oct4 interacted with PKM2 in NSCs and that no or little interactions could be observed in glioma spheroids (Figure 3b). DCA augmented the amount of Oct4 co-immunoprecipitated with PKM2 in NSCs and in glioma spheroids in an even larger extent (Figure 3b). Duolink, a Proximity Ligation Assay (PLA) (http://www.olink.com/products/duolink), confirmed that the interaction between Oct4 and PKM2 was a rare event in untreated glioma spheroids and that the number of interaction foci drastically increased upon DCA treatment (Figure 3c). To address the functional consequences of this PKM2–Oct4 interaction in glioma spheroids and in NSCs, we investigated the effect of DCA on a number of Oct4 transcriptional targets. As shown in Figure 3d, DCA decreased the expression of Taldo, Stat3, Sox2 and Gata6 in glioma spheroids but not in NSCs. We confirmed the DCA-induced downregulation of STAT3 and Sox2 using immunoblots (Supplementary Figure S7a). The latter results were surprising as it has been suggested that the Oct4–PKM2 interaction increased the transcriptional activity of Oct4.^34^ To support our results, we found that DCA decreased the recruitment of Oct4 to chromatin in glioma spheroids but not in NSCs (Figure 3e and Supplementary Figure S7b). These results were consistent with a DCA-induced interaction between PKM2 and Oct4, which resulted in a decrease in Oct4 transcriptional activity in glioma spheroids but not in NSCs.

**Figure 4:**
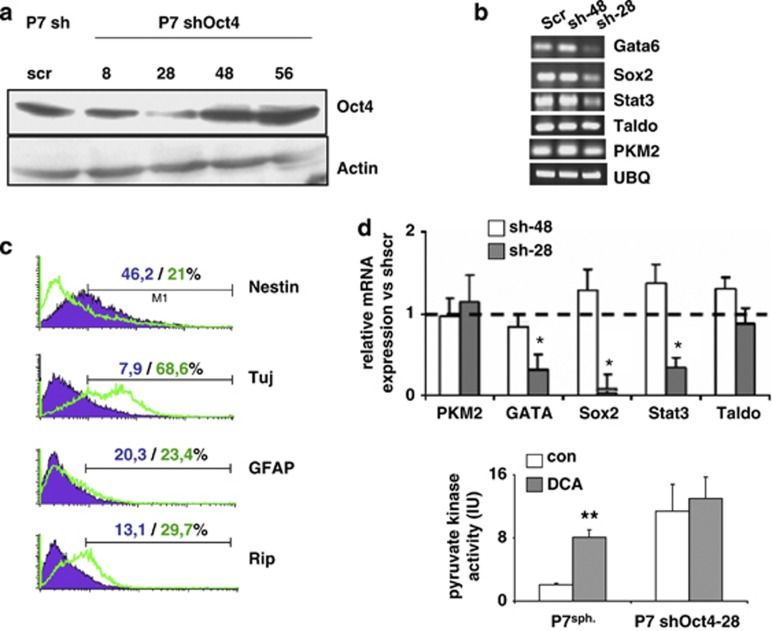
Oct4 downregulation induces glioma spheroids' differentiation and abrogates the effect of DCA on cell death. (**a** and **b**) The expression of PKM2 mRNA and of selected target genes of Oct4 was measured in glioma spheroids treated with either an efficient shRNA (sh-28) or an inefficient shRNA (sh-48). (**c**) The expression of the indicated markers was assessed using flow cytometry in glioma spheroids treated either with sh-scr or with an efficient shRNA directed against Oct4 (shRNA28). The graphs shown are representative of three independent experiments and the mean±S.D. percentage of positive cells is indicated. (**d**) Pyruvate kinase activity was measured in control and ShRNA Oct4-treated glioma spheroids in control and in DCA-treated (1 mM, 48 h) rat glioma spheroids (*P<0.05, **P<0.01)

To further evaluate the role of Oct4 in the regulation of PKM2, we downregulated the expression of Oct4 in glioma spheroids using a shRNA strategy. We used a shRNA that produced an 80% knockdown of Oct4 (Sh28) and, as a control, a shRNA that had no effect on Oct4 expression (Sh48) (Supplementary Figure S8). As expected, the knockdown of Oct4, even not complete, decreased its transcriptional activity (Figure 4a) and this was sufficient to induce glioma spheroid differentiation (Figures 4b and c). In addition, the knockdown of Oct4 enhanced PKM2 activity but no effect of DCA on this activity was observed in Sh-OCT4-treated cultures (Figure 4d).

### DCA induces human glioma spheroid apoptosis and differentiation via a PKM2–Oct4 interaction

**Figure 5:**
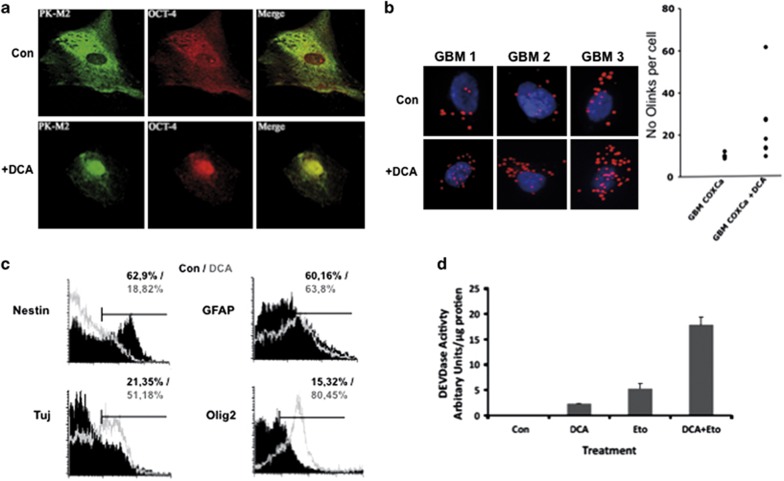
DCA sensitizes human glioma spheroids to apoptosis and induces their differentiation through PKM2–Oct4 interaction. (**a**) Confocal analyses of the subcellular localization of Oct4 and PKM2 in dissociated neurospheres. (**b**) PKM2–Oct4 complexes (red dots) were visualized by proximity ligation assay in control (Con) or DCA-treated human glioma spheroids and were quantified (graph). (**c**) The expression of the indicated markers was assessed using flow cytometry. The graphs shown are representative of three independent experiments and the mean percentage of positive cells is indicated. (**d**) Primary GBM cells were pretreated with 10 mM DCA for 24 h and then cultured in the absence or presence of 50 *μ*g/ml etoposide (Eto) for a further 24 h. The cell lysates were extracted and the caspase activity as determined by cleavage of the substrate DEVD was determined. The graph shown is representative of three independent experiments and the mean±S.D. is indicated

**Figure 6:**
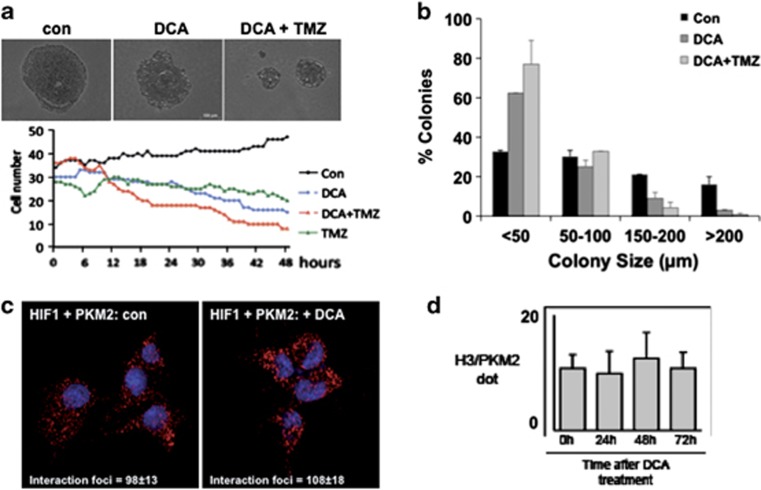
Effect of DCA on human GBM and specificity of the DCA-induced Oct4–PKM2 interaction. (**a**) Primary GBM cells were pretreated with 10 mM DCA for 24 h and then a time lapse was performed, in the absence or presence of 25 nM TMZ, over 48 h taking an image every 10 min. The cell number was determined for each hour. The data are representative of three individual experiments. (**b**) Primary GBM cultures were plated in soft agar in the presence or absence of DCA and TMZ. After 3 weeks, the size and the number of colonies were quantified. The data presented are the mean±S.D. of three different experiments. (**c**) The interaction between HIF and PKM2 was monitored by PLA/O-link at 5% O_2_ (hypoxia). (**d**) Similar experiments were performed at 20% O_2_ (normoxia) to analyze the interaction between Histone 3 (H3) and PKM2 in absence or in the presence of DCA

Next, we investigated the effect of DCA on several primary human glioma spheroids derived from *GBM* patients. As shown in Figure 5a, the addition of DCA induced the formation of PKM2–Oct4 complexes. However, contrary to rat glioma spheroids, an important fraction of the Oct4 could be found in both the cytoplasm and the nuclei, and the addition of DCA induced a major translocation of both Oct4 and PKM2 in the nuclei (Figure 5a). As in rat glioma spheroids, a treatment with DCA markedly increased the number of interactions between PKM2 and Oct4 as assessed by PLA (Figure 5b). We also confirmed that DCA induced the expression of neural differentiation markers in human glioma spheroids resulting in a downregulation of nestin and the upregulation of GFAP, beta-tubulin or olig2 (Figure 5c). DCA-induced cell death *per se*, quantified by a caspase 3 activity (DEVDase), was superior to that observed in rat cultures, a result consistent with previous reports^26, 27^ (Figure 5d). However, we observed that DCA synergized with Etoposide, a topoisomerase inhibitor, to induce cell death (Figure 5d). We also observed that the cells exhibited the same morphological modifications as those detected in rat glioma spheroid cultures, and these changes in morphology were observed even in the presence of Temozolomide (TMZ), the principal agent used as chemotherapy agent in GBM (Figure 6a). Moreover, the co-treatment of human glioma spheroids with DCA plus TMZ augmented the extent of cell death as compared with DCA alone (Figure 6a). Anchoring-independent growth was tested by growing cells in soft agarose. DCA did not only reduce the percentage of human glioma spheroid colonies but also the size of the colonies (Figure 6b). This latter point suggests that cell proliferation was reduced in DCA-treated colonies, a result compatible with a growth arrest due to cell differentiation. A similar but more drastic effect was observed with DCA plus TMZ (Figure 6b). Recently, PKM2 has been shown to interact with several nuclear proteins such as HIF-1^35^ and Histone H3.^18^ Hypoxia is commonly observed in gliomas and it has been suggested that it was regulating self-renewal and multipotency of CSC as well as glucose metabolism.^22^ We thus examined the effect of DCA on the HIF-1: PKM2 interaction under hypoxic conditions (that is, 5% O_2_). As illustrated in Figure 6c, Duolink analyses indicate that the addition of DCA affected neither the amount of interaction foci between HIF-1 and PKM2 nor their subcellular localization. Similarly, under normoxia (for example, 20% O_2_), we did not observe either *in vivo* (for example, for rat tumors) or *in vitro* (for example, human glioma cultures) an effect of DCA on PKM2–H3 interaction (Figure 6d) or on H3 phosphorylation (Supplementary Figure S9).

## Discussion

Over the past few years, our view on the role of PKM2 in cancer progression has evolved from a strictly metabolic function to a more multifaceted role, which includes the regulation of cell proliferation and cell death through transcriptional regulation. These different functions have been linked to the subcellular localization of PKM2, a mostly cytosolic protein, which can be translocated into the nuclei under some circumstances. In the nuclei, PKM2 exhibits a nuclear protein kinase activity and/or selectively binds to transcription factors or to histones and thus deeply affects cancer cell fate.^14, 36^ PKM2 under its dimeric form exhibits low PK activity that allows the accumulation of glycolysis intermediates available to sustain tumor growth by providing substrate for amino acids, lipids and nucleic acid precursors and therefore supporting cancer cell growth.^14^ A major result has been the identification of PKM2 as a binding partner of many tyrosine-phosphorylated peptides and as such as a potential regulator of many signaling pathways. The involvement of PKM2 in a growing list of different pathways relies mostly on protein–protein interactions and nuclear transport and implies nonanabolic metabolism, even though these pathways are not yet completely explored.^20^

Here we show that in adult glioma spheroids, the interaction of PKM2 with Oct4 inhibited its role in maintaining ‘stemness' in glioma stem cells thereby promoting differentiation and as such enhancing the sensitivity of these cells to cell death. Differentiation strategies have proven to be efficient in enhancing the glioma spheroid sensitivity to apoptosis and in reducing tumorigenicity.^9, 10, 11, 12^ These differentiating agents activated various signaling pathways, thereby inducing various differentiated phenotypes and all resulting in decreased tumorigenicity.^9, 10, 11, 12^

**Figure 7:**
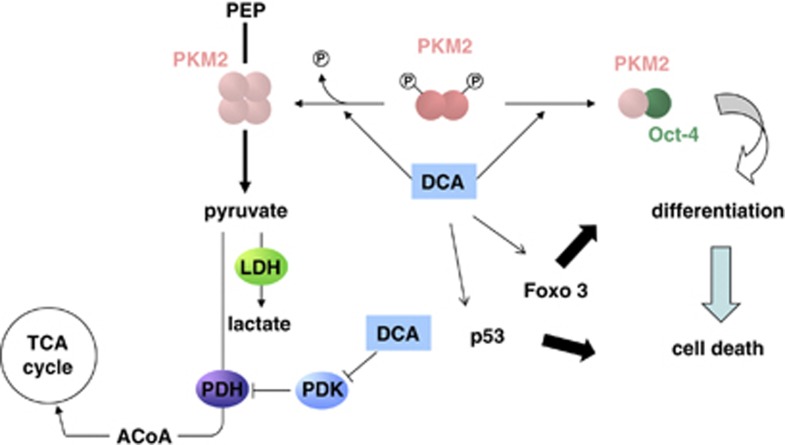
Schematic representation of the role of DCA in PKM2:Oct4-controlled glioma spheroids' differentiation and death. PKM2 activity is controlled by its substrate and oscillates between a dimeric (low activity) form and a tetrameric (high activity) form. The tetrameric form of PKM2 that produces pyruvate from phosphoenolpyruvate (PEP) is the main form in normal differentiated tissues and proliferating cells. In tumor cells, PKM2 is mainly dimeric and dimers are induced by the interaction with several oncogenes. Here, we show that Dichloroacetate induce both the tetrameric form and the interaction with Oct4. This interaction is responsible for the decrease in the transcription activity of Oct4 and thus induces the differentiation of glioma spheroids. The latter process is accompanied by an increase in the sensitivity of CSC to apoptosis. We have previously shown that DCA also sensibilizes glioma spheroids to apoptosis by modifying the expression of members of the Bcl-2 family and via the induction of p53 and FoxO3a activities (Morfouace *et al.*^21^). In addition to PKM2–Oct4 interaction, both FoxO3a and p53 can have additional roles in the differentiation of glioma spheroids

The Oct4–PKM2 interaction occurs concomitantly with a shift from the high-phosphorylated form of PKM2 (related to the dimer, low-activity conformation) to the low-phosphorylated PKM2 (related to the tetramer, high-activity complex). The mechanism regulating this interaction remains to be investigated. Whether the phosphorylation of PKM2 directly regulates the interaction is still unknown. Of note, PKM2 Y105, the phosphorylation of which regulates the shift between the dimer and the tetramer conformations^29^ is not included in the PKM2–Oct4 interaction site described by Lee *et al.*;^34^ however, we cannot exclude that this residue is implicated in the regulation of this interaction. Thus, based on our results and those published, we postulate that PKM2 can act both as a proliferative agent via its interaction with nuclear proteins or as a differentiating agent via its interaction with Oct4. Of note, under our conditions, we did not observe any modification of the phosphorylation of H3T11 (Supplementary Figure S8), a substrate of PKM2 protein kinase activity. It is tempting to assume that a balance between the oligomerization states of PKM2 could direct the tumor toward proliferation or differentiation independently of the activity (Figure 7).

In summary, we show that DCA could be a valuable adjuvant therapy for GBM patients, especially in tumors with a large CSC population but needs to be associated with another anticancer drug.

## Materials and Methods

### Materials

Cell culture material was obtained from Gibco (Life Technologies, Cergy Pontoise, France). Unless stated otherwise, all chemicals were purchased from Sigma-Aldrich (St. Louis, MO, USA). The references of antibodies and the dilutions used are indicated in Supplementary Data. Results shown are the mean values of at least three independent experiments (±S.D.) unless expressively mentioned. The ImageJ software (freely available online rsbweb.nih.gov/ij/) was used to quantify images.

### Cell culture

Glioma primary cultures (named P7 and M7) were obtained from Sprague–Dawley rats following antenatal ENU induction as described in Morfouace *et al.*^21^ Cells were either maintained in FCS-containing medium as adherent cells or cultured as neurospheres in FCS-free defined medium (DMEM 1 g/l glucose, 2 mM L-glutamine, N2- and B27-supplement, 2 *μ*g/ml heparin, 20 ng/ml EGF and 25 ng/ml bFGF, 100 U/ml penicillin and 100 *μ*g/ml streptomycin). Adult NSCs were obtained from 7-week-old Sprague–Dawley rat. Brains were dissected; SVZ was cultured according to the Stem Cell technologies protocol in defined medium. DCA (1 mM) was added in the cell culture when indicated.

Human primary GBM cultures were grown in defined medium (DMEM/Ham F12, 2 mM L-glutamine, N2 and B27 supplement, 2 *μ*g/ml heparin, 20 ng/ml EGF and 25 ng/ml bFGF, 100 U/ml penicillin and 100 *μ*g/ml streptomycin).

### 3D culture

Primary GBM cells (5 × 10^3^) resuspended in 0.35% soft agar containing the different compounds were layered on 0.5% agar. The soft agar layer was covered with media containing the compound to be tested. After 3 weeks, the cultures were scanned using a Leica DMI6000B and the Metamorph program.

### RT and qPCR

Cells were washed twice in PBS, and then total RNA was isolated using the RNeasy MiniKit (Qiagen, Courtaboeuf, France) following the manufacturer's instructions with DNAse I treatment. After RNA quantification using the Nano Drop (Nano Drop ND-1000, Thermo Fisher Scientific, Waltham, MA, USA), 1 *μ*g RNA was reverse-transcribed using Reverse Transcriptase AffinityScript (Agilent-Stratagene, Massy, France) for cDNA synthesis. Quantitative real-time PCR assays were performed and monitored in triplicate using an MX4000 multiplex Quantitative PCR system (Agilent-Stratagene).

### Protein extraction and immunoblotting

Total proteins were extracted in 1% NP-40, 0.5% sodium-deoxycholate, 0.1% SDS supplemented with protease inhibitor cocktail from Roche Diagnostics (Mannheim, Germany). The proteins linked to the chromatin were extracted as described in Hervouet *et al.*^38^ Protein concentration was determined using Bradford assay (Bio-Rad, Hercules, CA, USA). Protein extracts were separated by SDS-PAGE, transferred onto PVDF membrane (Millipore, St. Quentin-Yvelines, France) and revealed with ECL (Roche Diagnostics). HRP-conjugated secondary antibodies were from Bio-Rad.

### LDH activity measurement

Cells were plated at a density of 10^4^ cells/100 *μ*l in 96-well plates. The cells were lysed by the addition of 10 *μ*l lysis solution incubated at 37 °C for 45 min. Fifty microliter supernatant were used to determine the LDH concentration according to the manufacturer's instructions (Promega, Charbonnières, France).

### Tumor xenograft

A total of 5 × 10^4^ glioma cells (P7 primary culture) cultured in defined medium were injected subcutaneously into the flank of male nude mice. Mice were evaluated twice a week over a 3-week period. Tumor volume was measured with a caliper. When indicated, DCA was added in the drinking water and renewed twice a week from the injection day (0.75 g/l, corresponding to an average ingestion of 80 mg/kg per day (Morfouace *et al.*^21^). After 37 days, tumors were removed, included in a paraffin block and sliced. Immunostaining was performed as described below for neurospheres.

### Flow cytometry

Cells were dissociated, washed and marked as described by Sergent-Tanguy *et al.*^37^ Data acquisition was performed on a FACScalibur (Becton Dickinson, Le Pont-de-Claix, France). Data were analyzed using the CellQuest software (Becton Dickinson).

### Immunostaining

For immunostaining, neurospheres were dissociated and 50 *μ*l were spotted onto Superfrost slides. The cells were then fixed with 4% paraformaldehyde for 20 min, permeabilized with 0.1% SDS for 10 min, blocked with 3% BSA for 20 min and incubated with primary antibody for 1H followed by Alexa568-coupled secondary antibody incubation (Molecular Probes, Eugene, OR, USA) for 1H. Cells were mounted in a medium containing Dapi (Life Technologies) to visualize nuclei. The staining was detected by apotome microscopy (Zeiss Axiovert 200-M inverted microscope and AxioVision 4.6 program, Carl Zeiss Gbmh, Oberkochen, Germany).

### PK activity

PK activity was measured using the Pyruvate Kinase Assay Kit from BioVision (Milpitas, CA, USA). The enzymatic activity was normalized to protein concentration assessed by BCA.

### Immunoprecipitation

Total proteins were extracted in CHAPS buffer (10 mM Hepes, 150 mM NaCl, 1% CHAPS, pH 7.4 and EDTA-free protease inhibitors cocktail) as previously described and were immunoprecipitated with the Catch and Release kit, as described by the manufacturer (Millipore).

### Proximity ligation assay

Neurospheres were mechanically dissociated and 50 *μ*l were spotted onto Superfrost slides. The cells were fixed in 4% paraformaldehyde for 20 min, permeabilized with 0.25% Triton-PBS for 30 min and then treated according to the manufacturer's instructions (Olink Bioscience, Uppsala, Sweden). Cells were mounted in a medium containing Dapi (Life Technologies) to visualize the nuclei. The staining was detected with apotome microscopy (Zeiss Axiovert 200-M inverted microscope and AxioVision 4.6 program).

## Abbreviations

CSCs: Cancer stem cells
DCA: Dichloroacetate
ENU: ethylnitrosourea
NSCs: neural stem cells
Oct4: octamer-binding transcription factor 4
PKM2: pyruvate kinase muscle isozyme 2
