# Supplementary material for: Control of glioma cell death and differentiation by PKM2–Oct4 interaction
Source: Cell Death Dis. 2014 Jan 30;5(1):e1036–. doi: 10.1038/cddis.2013.561 (PMC4040711; doi:10.1038/cddis.2013.561)
Supplement: Supplementary Information [file cddis2013561x1.ppt]

## Slide 1
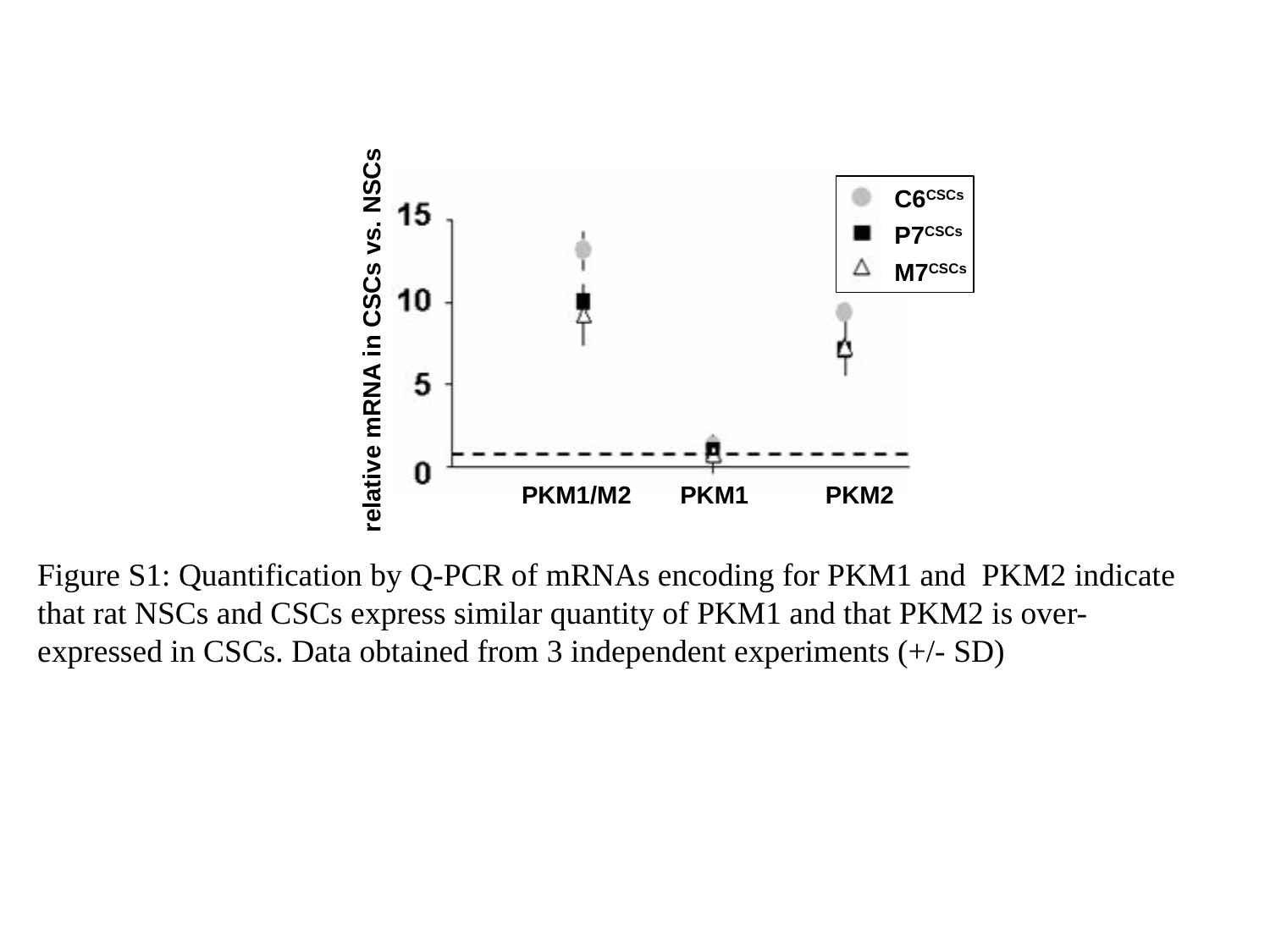

C6CSCs
P7CSCs
M7CSCs
relative mRNA in CSCs vs. NSCs
PKM1/M2 PKM1 PKM2
Figure S1: Quantification by Q-PCR of mRNAs encoding for PKM1 and PKM2 indicate that rat NSCs and CSCs express similar quantity of PKM1 and that PKM2 is over-expressed in CSCs. Data obtained from 3 independent experiments (+/- SD)

## Slide 2
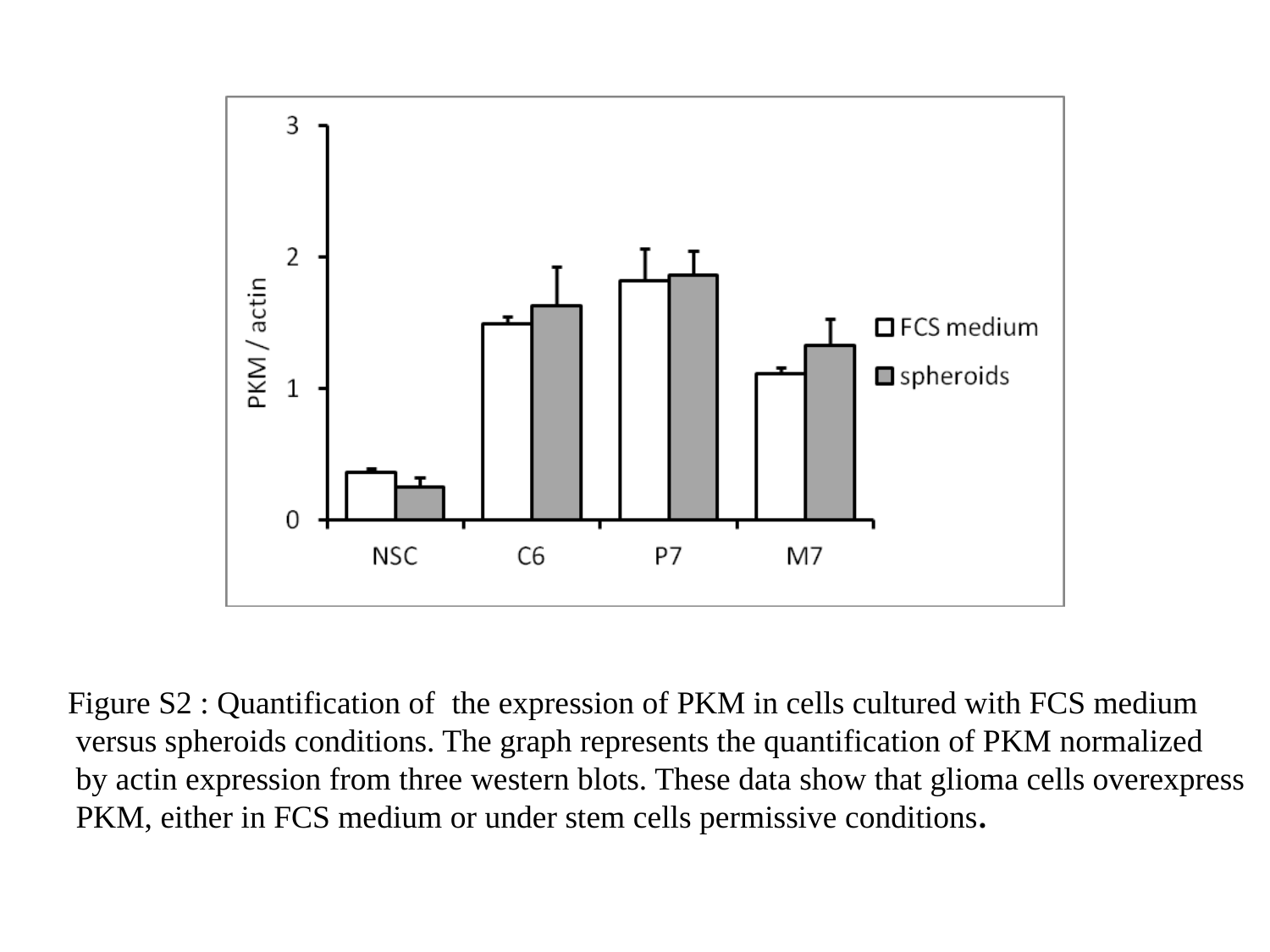

Figure S2 : Quantification of the expression of PKM in cells cultured with FCS medium
 versus spheroids conditions. The graph represents the quantification of PKM normalized
 by actin expression from three western blots. These data show that glioma cells overexpress
 PKM, either in FCS medium or under stem cells permissive conditions.

## Slide 3
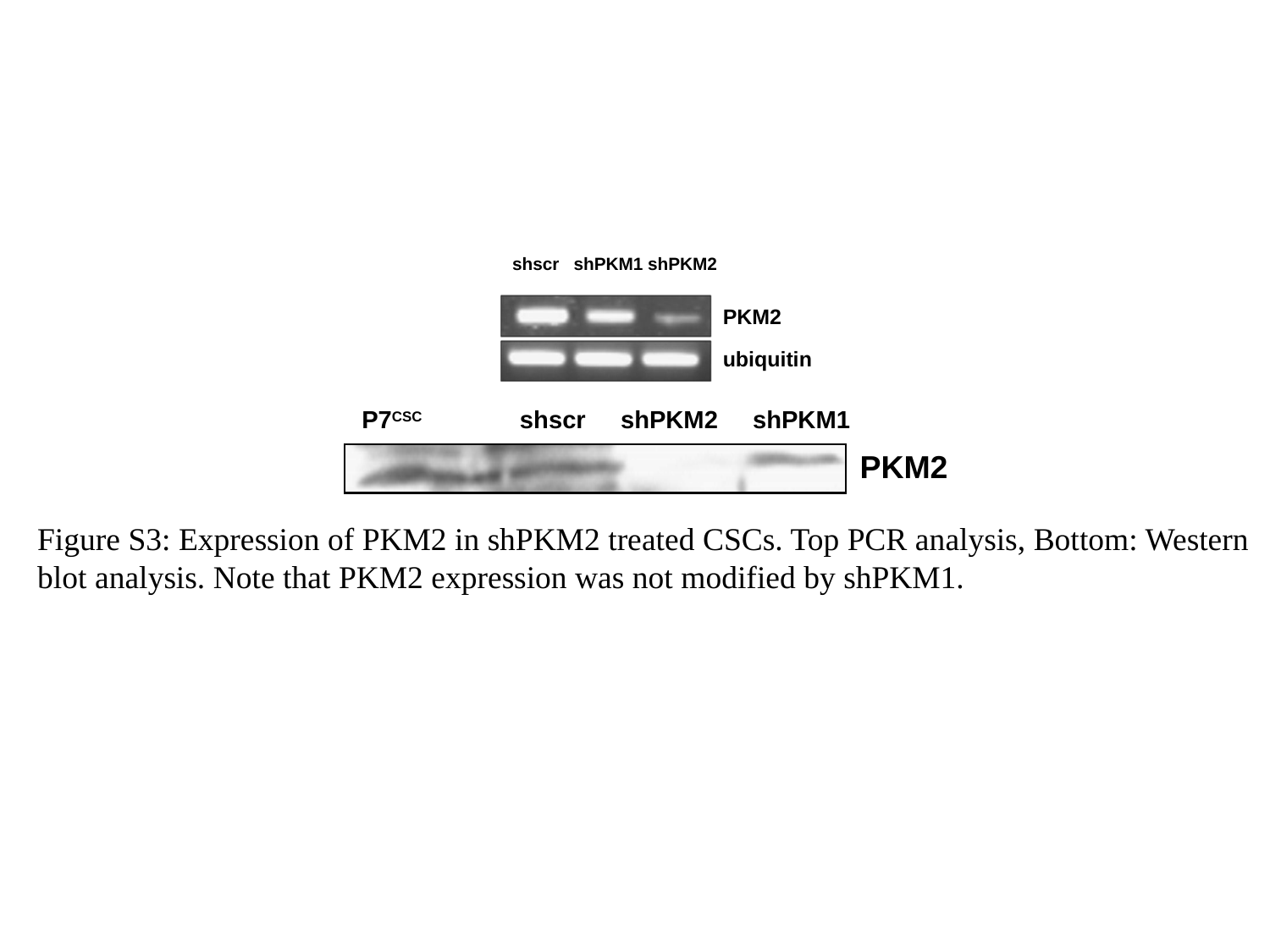

shscr shPKM1 shPKM2
PKM2
ubiquitin
P7CSC shscr shPKM2 shPKM1
PKM2
Figure S3: Expression of PKM2 in shPKM2 treated CSCs. Top PCR analysis, Bottom: Western blot analysis. Note that PKM2 expression was not modified by shPKM1.

## Slide 4
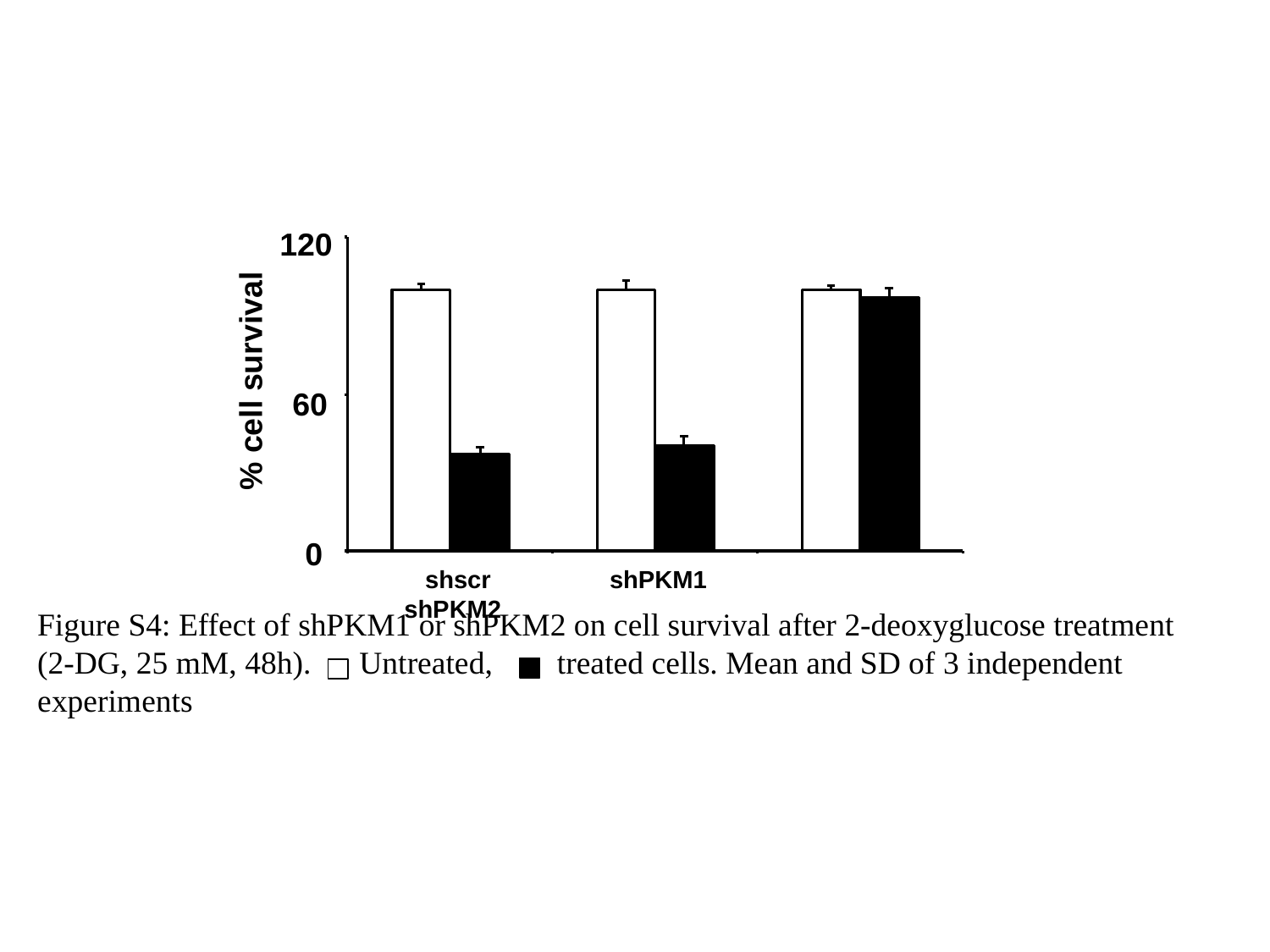

120
% cell survival
60
0
 shscr shPKM1 shPKM2
Figure S4: Effect of shPKM1 or shPKM2 on cell survival after 2-deoxyglucose treatment
(2-DG, 25 mM, 48h). Untreated, treated cells. Mean and SD of 3 independent experiments

## Slide 5
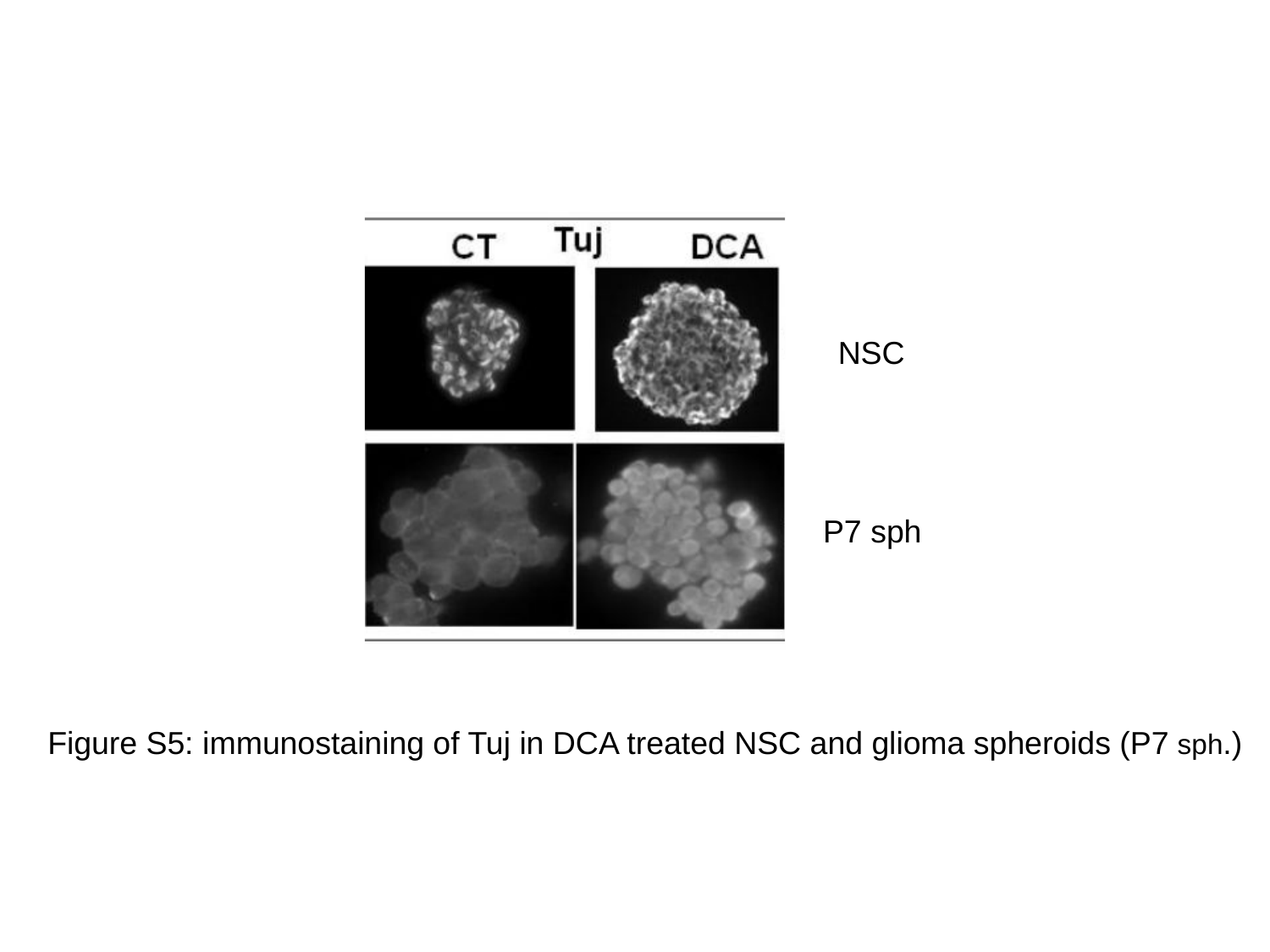

NSC
P7 sph
Figure S5: immunostaining of Tuj in DCA treated NSC and glioma spheroids (P7 sph.)

## Slide 6
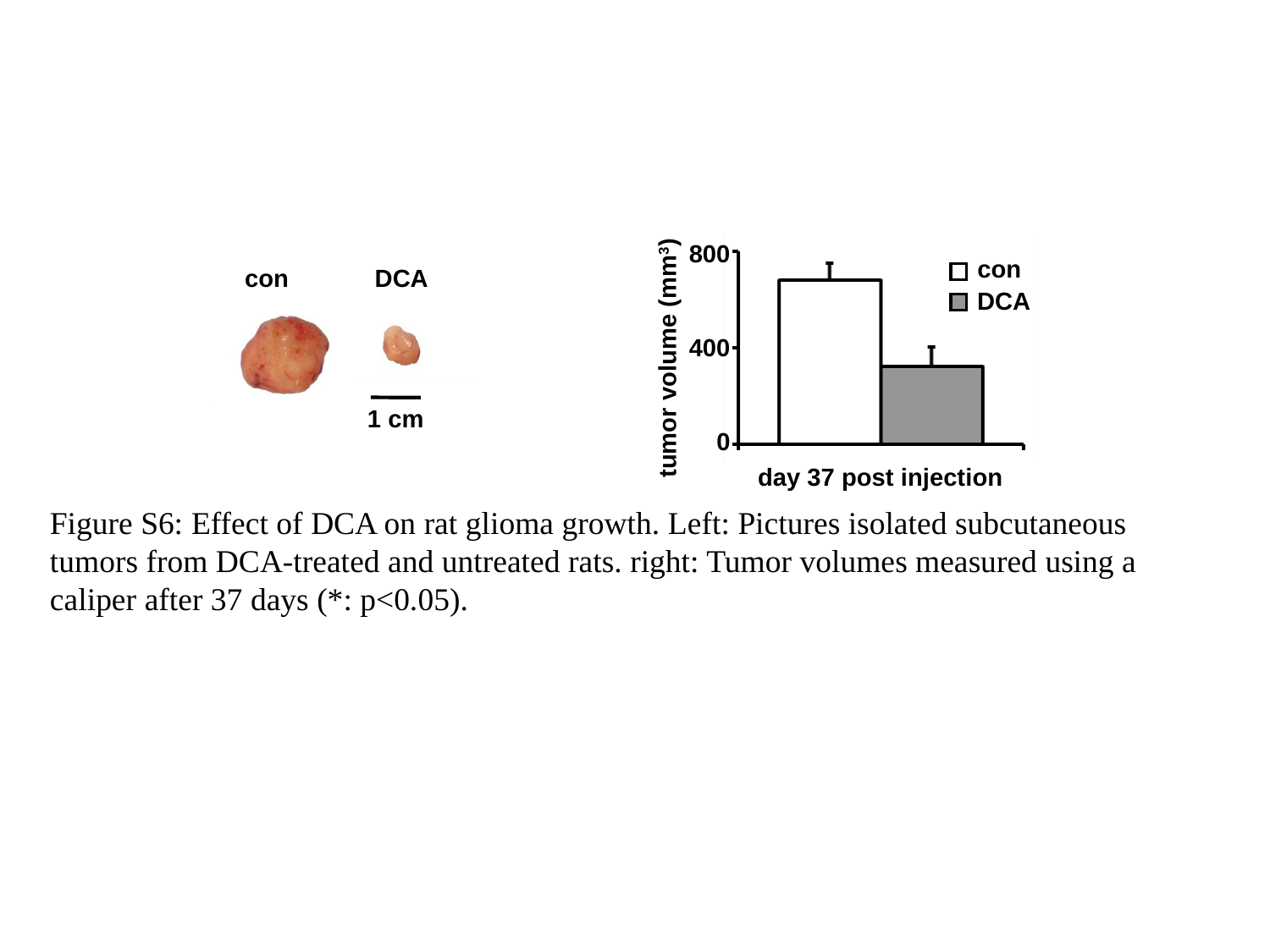

800
con
DCA
400
tumor volume (mm3)
0
day 37 post injection
con
DCA
1 cm
Figure S6: Effect of DCA on rat glioma growth. Left: Pictures isolated subcutaneous tumors from DCA-treated and untreated rats. right: Tumor volumes measured using a caliper after 37 days (*: p<0.05).

## Slide 7
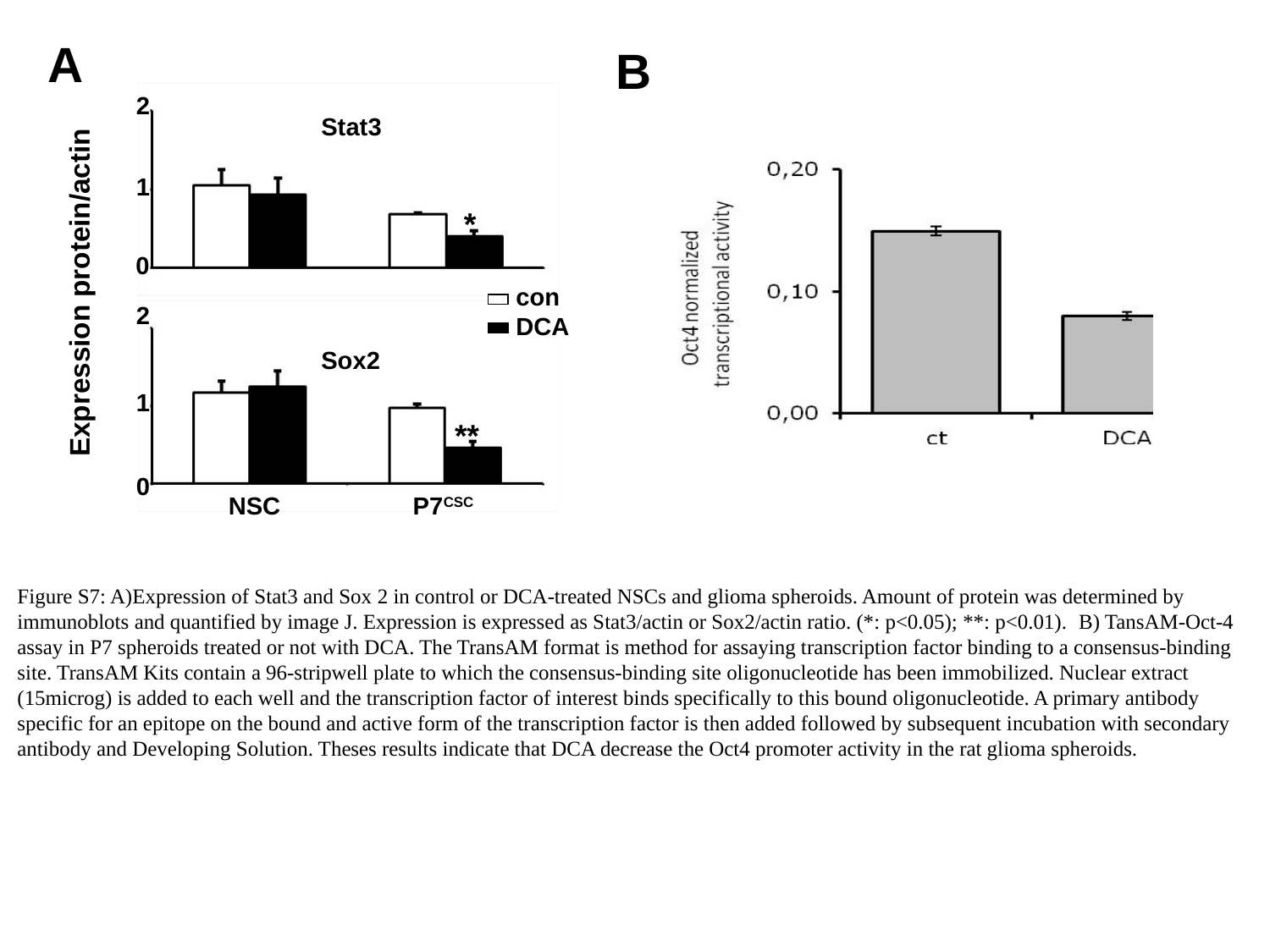

A
B
2
Stat3
1
*
0
Expression protein/actin
con
DCA
2
Sox2
1
**
0
NSC P7CSC
Figure S7: A)Expression of Stat3 and Sox 2 in control or DCA-treated NSCs and glioma spheroids. Amount of protein was determined by immunoblots and quantified by image J. Expression is expressed as Stat3/actin or Sox2/actin ratio. (*: p<0.05); **: p<0.01).  B) TansAM-Oct-4 assay in P7 spheroids treated or not with DCA. The TransAM format is method for assaying transcription factor binding to a consensus-binding site. TransAM Kits contain a 96-stripwell plate to which the consensus-binding site oligonucleotide has been immobilized. Nuclear extract (15microg) is added to each well and the transcription factor of interest binds specifically to this bound oligonucleotide. A primary antibody specific for an epitope on the bound and active form of the transcription factor is then added followed by subsequent incubation with secondary antibody and Developing Solution. Theses results indicate that DCA decrease the Oct4 promoter activity in the rat glioma spheroids.

## Slide 8
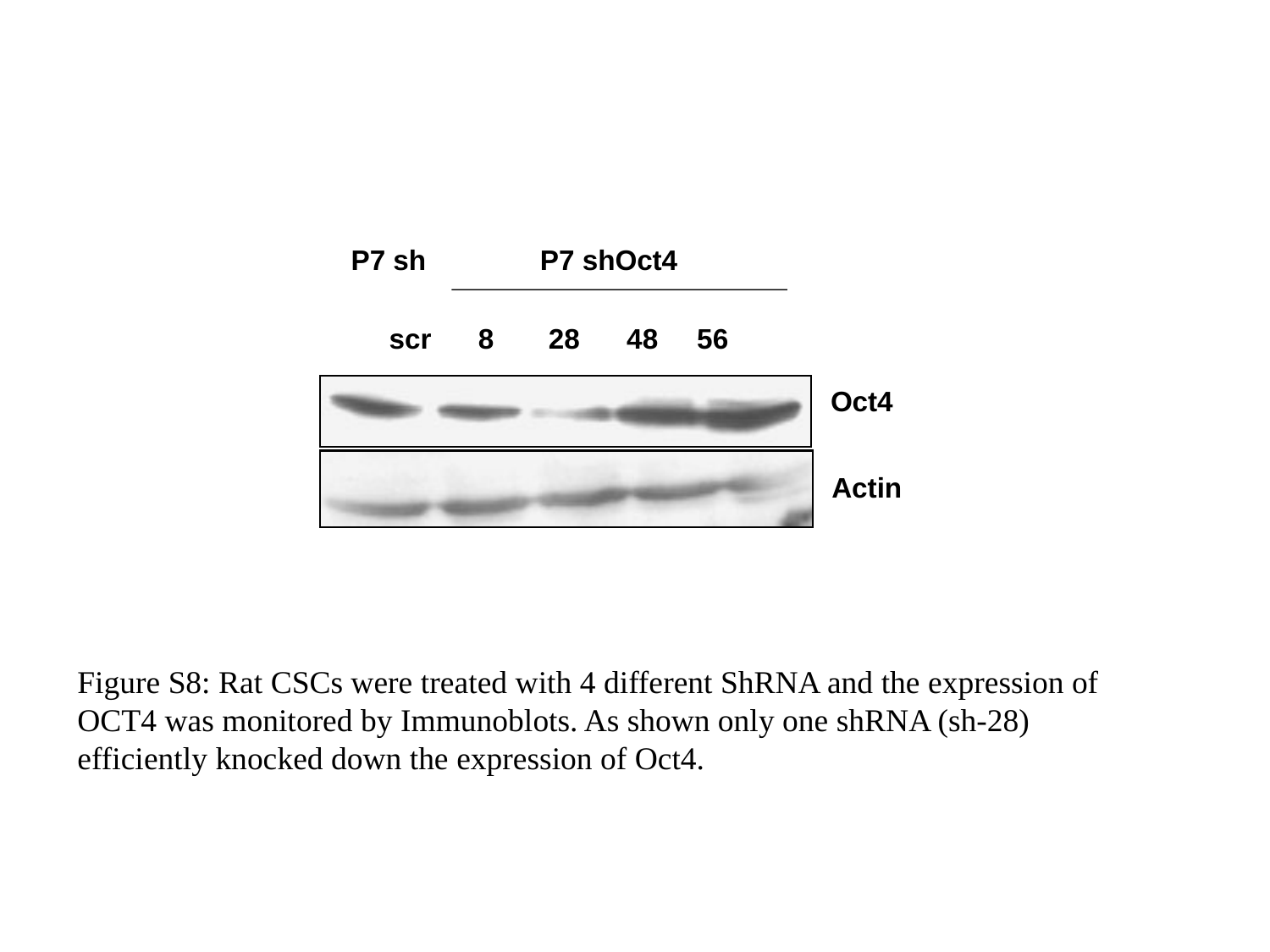

P7 sh
P7 shOct4
 scr 8 28 48 56
Oct4
Actin
Figure S8: Rat CSCs were treated with 4 different ShRNA and the expression of OCT4 was monitored by Immunoblots. As shown only one shRNA (sh-28) efficiently knocked down the expression of Oct4.

## Slide 9
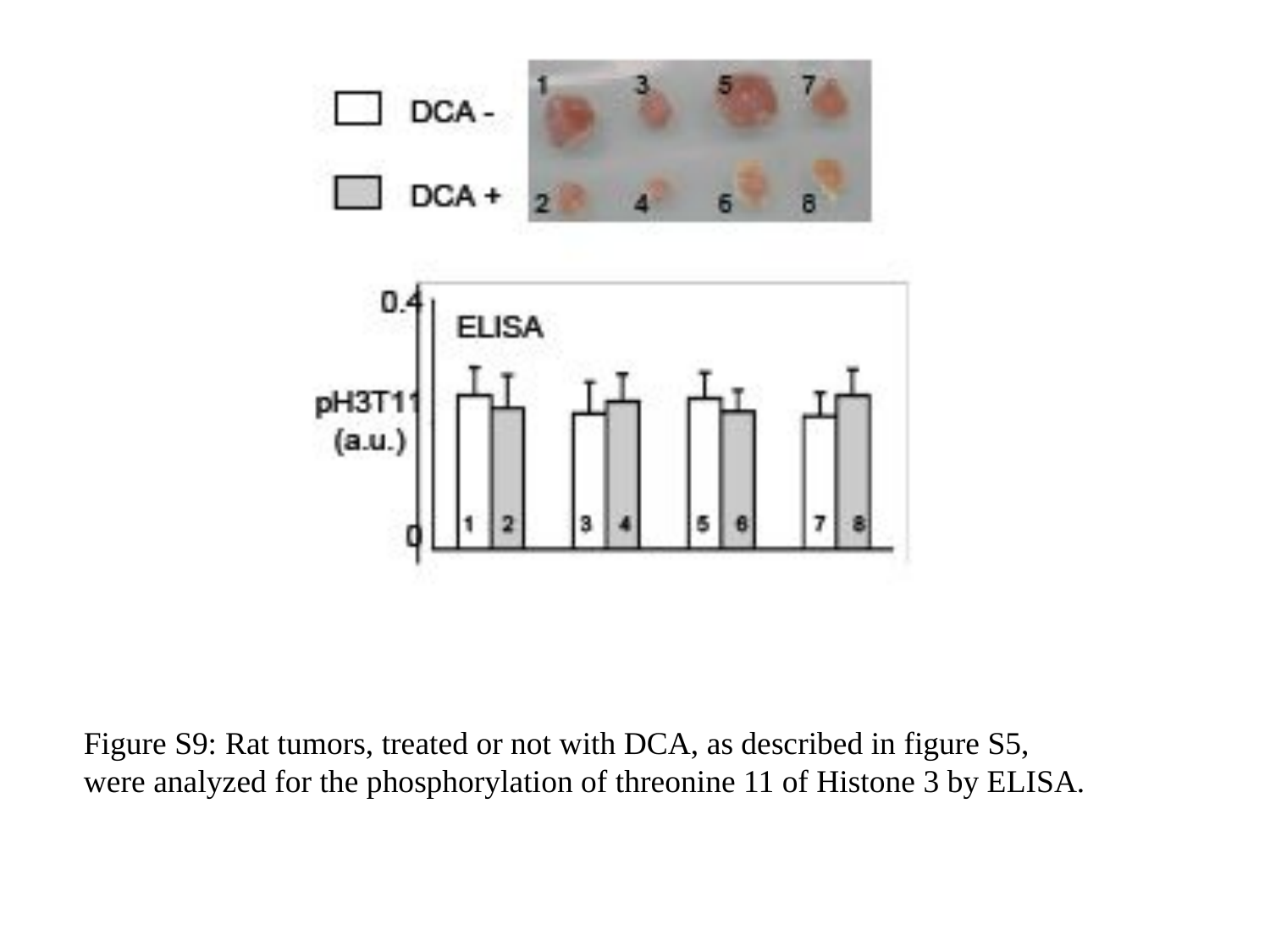

Figure S9: Rat tumors, treated or not with DCA, as described in figure S5,
were analyzed for the phosphorylation of threonine 11 of Histone 3 by ELISA.
